# Nuclear Export Inhibition for Pancreatic Cancer Therapy

**DOI:** 10.3390/cancers10050138

**Published:** 2018-05-07

**Authors:** Irfana Muqbil, Asfar S. Azmi, Ramzi M. Mohammad

**Affiliations:** 1 Department of Chemistry, University of Detroit Mercy, Detroit, MI 48221, USA; muqbilr@udmercy.edu; 2 Department of Oncology, Karmanos Cancer Institute, Wayne State University School of Medicine, Detroit, MI 48201, USA; azmia@karmanos.org

**Keywords:** Pancreatic Cancer, Pancreatic Ductal Adenocarcinoma, nuclear protein export, Exportin-1, CRM1, Specific Inhibitors of Nuclear Export, SINE, Phase I

## Abstract

Pancreatic cancer is a deadly disease that is resistant to most available therapeutics. Pancreatic cancer to date has no effective drugs that could enhance the survival of patients once their disease has metastasized. There is a need for the identification of novel actionable drug targets in this unusually recalcitrant cancer. Nuclear protein transport is an important mechanism that regulates the function of several tumor suppressor proteins (TSPs) in a compartmentalization-dependent manner. High expression of the nuclear exporter chromosome maintenance region 1 (CRM1) or exportin 1 (XPO1), a common feature of several cancers including pancreatic cancer, results in excessive export of critical TSPs to the incorrect cellular compartment, leading to their functional inactivation. Small molecule inhibitors of XPO1 can block this export, retaining very important and functional TSPs in the nucleus and leading to the effective killing of the cancer cells. This review highlights the current knowledge on the role of XPO1 in pancreatic cancer and how this serves as a unique and clinically viable target in this devastating and by far incurable cancer.

## 1. Introduction

Pancreatic cancer is soon going to be the 3rd leading cause of cancer-related deaths in the United States [1]. By 2030, it is estimated that the annual deaths from pancreatic cancer will overtake that from colon and liver cancers combined (https://www.cancer.org/cancer/pancreatic-cancer/about/key-statistics.html). Pancreatic cancer has one of the worst prognoses, with a five years survival at ~7–8%. A very small fraction of patients are eligible for surgical resection at the time of diagnosis. This makes chemotherapy the only option for the majority of patients once their disease has metastasized. Pancreatic cancer acutely lacks early detection markers and specific therapeutic targets. Tumor cells sit in a milieu of several cellular types such as stromal cells, stellate cells, and induced pluripotent cells that together form a very sophisticated tumor microenvironment [2]. The presence of dense stroma prevents proper drug penetrance to the tumor site [3]. Simultaneously, the microenvironment of pancreatic tumors is immunologically privileged consisting of regulatory T cell (Tregs) tumor-associated macrophages (TAMs), and myeloid-derived suppressive cells (MDSCs), that block proper anti-tumor immune activation and also render most of the immunotherapy strategies ineffective [4]. The presence of a resistant population of cells (cancer stem-like cells or CSCs) in the pancreatic tumors has also been attributed to therapy resistance and disease recurrence [5].

Significant molecular work over the past three decades has unraveled the mechanisms guiding the development and progression of pancreatic cancer [6]. Common alterations include the presence of K-ras mutations (>80–90%) and inactivation of tumor suppressors such as CDKN2A, p53, BRCA2 and SMAD4 [7]. Despite frequent alterations in these four tumor suppressor genes, it is surprising to note that most of the remaining tumor suppressors to a large extent remain wild type [8]. However, these Tumor Suppressor Protein (TSPs) are found to be dysfunctional due to mislocalization in the event of increased XPO1 expression and cannot be activated even in the presence of chemotherapeutics or targeted drugs. There are several post-translational mechanisms that block the activation of TSPs [9,10]. Emerging studies show that a significant proportion of TSPs and genome surveillance proteins are inactivated through mislocalization which is governed by nuclear import and export signaling. More significantly, the aberrant nuclear protein export is commonly observed in the majority of cancers including pancreatic cancer. This has been implicated in rendering TSPs dysfunctional. Therefore, relocalization of these important TSPs with low mutational frequency to the right cellular compartment by blocking nuclear export becomes an attractive therapeutic strategy in pancreatic cancer.

## 2. Nuclear Protein Transport

With the introduction of sub-cellular organelles in eukaryotes came the need for efficient transport mechanisms for exchange of biological material across their membranous enclosures [11]. Eukaryotic cells have evolutionarily adapted to exchange biological material across the nuclear membrane and other cellular membranes. The nuclear membrane is a double membrane surrounding the nucleus. Ions and biological entities (<40 KDa) can diffuse freely across the nuclear membrane. Such transport occurs through a simple diffusion gradient. However, molecules, proteins and RNA (>40 KDa) require an active transport system to cross the nuclear membrane barrier [12]. Such transport happens through specialized gated machinery or nuclear pore complex [13]. Nuclear pores are formed from large protein complexes that traverse the nuclear envelope. Each mammalian cell has more than 2000 nuclear pore complexes and more than 500 proteins (that make up the nuclear pore complex) known as nucleoporins.

**Figure 1:**
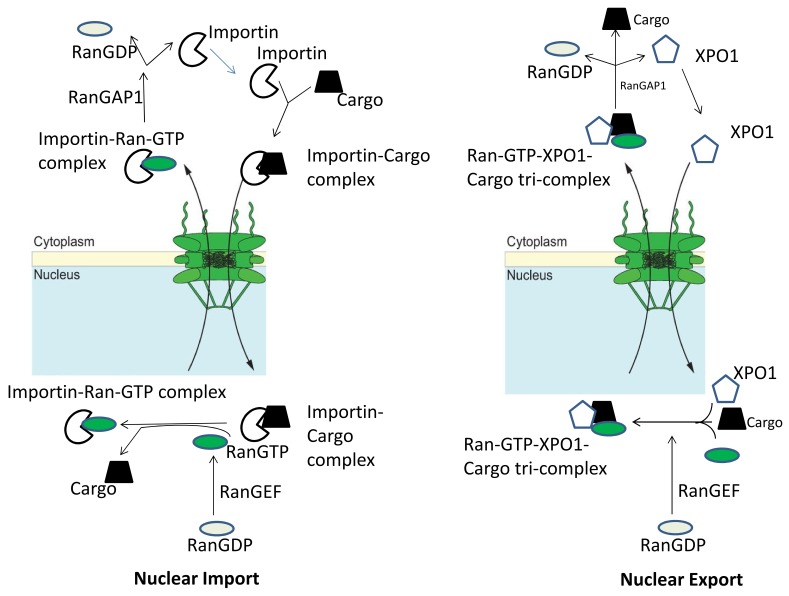
Nuclear transport machinery. An import complex consisting of a nuclear localization signal (NLS)-bearing cargo and an Importin is formed in the cytoplasm. After translocation through the nuclear pore complex, Ran-GTP displaces the cargo from the importin, resulting in nuclear cargo release. The importin–Ran-GTP complex returns to the cytoplasm through the Nuclear Pore Complex (NPC) where the Ran GTPase-activating protein (RanGAP1) stimulates GTP hydrolysis, releasing the Nuclear Transport Receptor (NTR) for another import cycle. Nuclear export cycles require the formation of a trimeric cargo–XPO1–Ran-GTP complex in the nucleus. After NPC passage, this complex dissociates due to Ran-GTP hydrolysis, releasing the cargo into the cytoplasm. This figure is adopted from the figure in the article by Marion Weberruss and Wolfram Antonin [25]. This is an Open Access article distributed under the terms of the Creative Commons Attribution License (http://creativecommons.org/licenses/by/3.0), which permits unrestricted use, distribution and reproduction in any medium provided that the original work is properly attributed.

Another important component is the carrier proteins belonging to the Karyopherin family. They are responsible for shuttling nuclear proteins to the cytosol and vice versa in an energy-dependent process that is facilitated by the small Ras-related GTPase RAN gradient [14]. There are several proteins in this large Karyopherin family (genes encoded by Karyopherin are KPNA1, KPNA2, KPNA3, KPNA4, KPNA5, KPNA6, KPNB1, and CRM1). Cargo protein recognition is aided by the presence of nuclear localization signal (NLS) or nuclear export signal (NES) [15]. NLS is a short sequence of positively charged lysines or arginines exposed on the protein surface [16]. On the other hand, NES is a short amino acid sequence of 4 hydrophobic amino acids (arranged as L_xxx_L_xx_L_x_L where L is a leucine) found in most proteins that targets it for export to the cytoplasm [17]. Importins recognize the NLS sequence in target proteins [18], while exportins [particularly exportin 1 (XPO1) also known as chromosome maintenance region 1/CRM1] sequester cytosolic proteins from the nucleus through NES recognition (Figure 1) [19]. A coordinated nuclear import and export helps the spatial regulation and function of several critical proteins in cells. Aberrations in import or export, as commonly observed in cancer cells have been shown to alter this balance leading to the functional inactivation of TSPs and other vital proteins.

### 2.1. Karyopherin Classification

#### 2.1.1. Importin Alpha

Importin α also referred to as karyopherin α is an adaptor protein that primarily plays a role in the import of proteins to the cell nucleus. The importin α acts as a facilitator for recognition and binding of the cargo protein to the importin β [20]. The presence of importin β binding domain at the N terminal of importin α helps its binding to the importin β. Importin β then transports the NLS-containing cargo protein across the nuclear envelope to the nuclear compartment. The interaction between importin α and importin β (importin α/β heterodimer) occurs only during the import process. Importin α also possess a cellular apoptosis susceptibility protein (CAS) binding domain [21]. Binding to the CAS allows the recycling of importin α back into the cytosol. This process is aided by the RAN GTP. Once in the cytosol, RAN GTP is hydrolyzed to RAN GDP resulting in the dissociation of importin α from CAS.

#### 2.1.2. Importin Beta

Importin-β are a group of proteins in the Karyopherin family and considered as major cytosolic protein importers [22]. Structurally they are composed of 19 tandem repeats of the HEAT motif (where two alpha helices are linked by a loop) [23]. In order to perform its nuclear import function, importin-β first associates to the nuclear pore complexes through weak and transient bonds with nucleoporins at their various Phe-Gly motifs that is facilitated by the importin α. This complex diffuses from the cytosol into the nucleus that is dependent on the concentration gradient i.e., the levels of importin α and GTP in the nucleus. Once inside the nucleus, the Ran GTPase enzyme hydrolyzes the GTP to GDP that results in changes in the binding affinity between importin β and the cargo thereby releasing the target in the nucleus [24]. Once the cargo is released, the association of the importin with CAS-Ran GTP recycles the importins back to the cytosol for the next round of import.

#### 2.1.3. Exportins

The function of exportins is opposite to that of importins. As the name suggests, exportins transport cargoes out of the nucleus to the cytosol. Although several different exportins are known, the chromosome maintenance region 1 (CRM1)/exportin 1 or XPO1 is recognized to be the major exporter of the majority of proteins, some mRNAs, rRNAs and snRNAs [19]. A similar protein Exportin 5 or XPO5 is quite well studied for its role in the export of precursor microRNAs that require cytosolic modifications for maturation [26]. XPO1 mediates the export of nuclear proteins through the recognition of a highly conserved nuclear export signal (NES). Recognition of XPO1 to NES is facilitated by RanGTP. The movement of cargo occurs due to the differences in the concentration gradient of RanGTP between the cytosol and nucleus. Once the complex reaches the cytosol, GTPase hydrolyzes the RanGTP to RanGDP. This results in the dissociation of the cargo from the XPO1 which is again ready to diffuse back to the nucleus to undertake the next round of export.

The biological importance of XPO1 expression was originally studied in developmental models of Xenopus and Drosophila in the 1980s [27]. In Xenopus, XPO1 is found to be expressed throughout their development, mostly residing in the nucleus although not capable of recognizing the NES. Similarly, studies in Drosophila model showed that the XPO1 counterpart termed embargoed (emb) to be also expressed through all the developmental stages [28]. In eukaryotic cells, XPO1 is considered the sole exporter of most of the tumor suppressor proteins. Presently there are more than 200 known export targets of XPO1. Most proteins carry the classical cognate peptide that is recognized by XPO1. NES predictors such as LocNES have been developed that query proteins with putative export signals http://prodata.swmed.edu/LocNES. It uses a supervised machine-learning algorithm to predict NESs in proteins. Recently, the diversity in NESs has been analyzed using bioinformatics approaches. NoLogo is a generative probabilistic model for predicting XPO1-dependent NESs. This platform led to the prediction that about 30% of NESs belong to distinct classes rather than the 6–10 classes proposed by existing models [29].

## 3. Exportin Expression in Pancreatic Cancer

**Figure 2:**
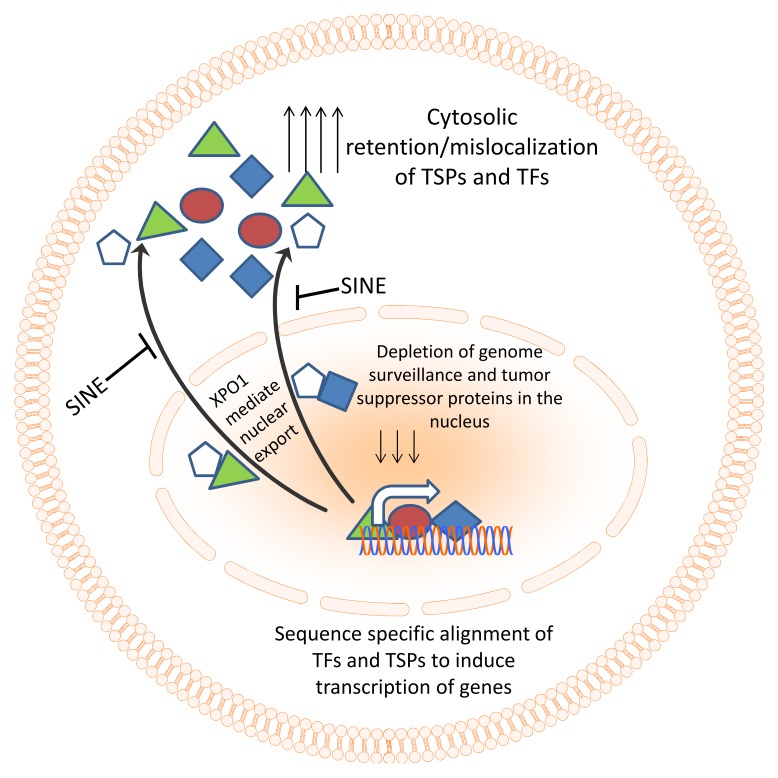
Nuclear export machinery as a therapeutic target. Genome surveillance and tumor suppressor proteins need to align to DNA in a sequence-specific manner in order to coordinate their surveillance and tumor suppressive function. In cancer, the excessive export of these TSPs results in their accumulation in the cytosol and functional inactivation. Specific inhibitors of nuclear export (SINE) compounds can block the nuclear export and restore TSPs in the nucleus of cancer cells leading to activation of tumor suppressive function.

Given its significance in regulating the expression of so many critical tumor suppressors and genome surveillance proteins, it is not surprising to note that XPO1 is found to be over-expressed in several different tumor types [30]. This review focusses on the impact of XPO1 over-expression in pancreatic cancer therapy resistance and how this can be harnessed for the development of effective therapeutics to tame this deadly disease (Figure 2).

The prognostic value of XPO1 in pancreatic cancer has already been established. In 2009 Huang and colleagues used 69 tumors and 10 normal tissues to demonstrate increased expression of XPO1 in pancreatic cancer [31]. More significantly, their studies showed that the high expression of XPO1 was associated with pronounced serum levels of CEA and CA19-9 in a statistically significant manner. Further, these authors showed a direct correlation between high XPO1 expression and tumor size, lymphadenopathy and liver metastasis. Their studies also confirmed the prognostic significance of XPO1 as an indicator for progression-free survival (PFS) as well as overall survival (OS) (both statistically significant). Higher expression of XPO1 was an independent prognostic parameter for poorer PFS and OS. Appraising this data, another study in 91 pancreatic patient tissues showed higher expression of XPO1 compared to that in matched normal control [32]. In a recent study, XPO1 was shown to be expressed in a significant proportion of pancreatic cancer, and increased expression correlates with both survivin expression and increased proliferative activity, suggesting that selective inhibitors of nuclear export may be effective against pancreatic cancer [33]. Collectively, work from several laboratories clearly indicates that XPO1 is aberrantly over-expressed in pancreatic cancer. Karyopherin family members are multi-faceted proteins with several biological functions. The family member XPO5 is a recognized exporter of precursor small non-coding RNAs (microRNAs). MicroRNAs exert their gene-regulatory effects by binding to the 3′ untranslated region of target mRNA, leading to degradation of the mRNA or inhibition of appropriate translation [34]. More importantly, it has been found that aberrations in specific miRNAs and their target genes promotes various cancer types including pancreatic cancer [35]. Studies have shown that aside from XPO5, the TSP exporter XPO1, in addition to its nuclear protein export function, can, in fact, export microRNAs as well [36]. Therefore, it is a fairly strong assumption that the alterations in the expression of XPO1 or its nuclear exporter activity may impact miRNA biogenesis [37]. Working in this direction we demonstrated that XPO1 RNAi interference or chemical inhibition by XPO1 specific small molecule drugs could re-align tumor suppressive microRNAs leading to inhibition of pancreatic cellular growth [38]. These studies and other investigations discussed in the forthcoming passage indicate to the key roles of XPO1 in PDAC subsistence pathways.

Despite the consistent observation showing enhancement in XPO1 expression in pancreatic cancer, the underlying mechanism for such activation is not clear. In an earlier study, the link to enhancement in human gonadotropin hormone hcg1 and XPO1 nuclear export activity activation has been described [39]. Interestingly, in supporting studies the enhancement in the expression of human gonadotropin enzyme has been linked to the pancreatic cancer development [40]. Regardless of these links, more work needs to be done to clearly demonstrate the association between the pregnancy hormone and XPO1 expression and nuclear export function enhancement in cancer. Our analysis of pancreatic cancer cell lines, pancreatic cancer stem cells (derived from pancreatic cancer cell lines) showed enhancement in XPO1 expression [41]. RNAi of XPO1 blocked pancreatic cancer cell line growth and also inhibited pancreatic cancer stem cell-derived spheroid formation indicating to their role in this devastating disease.

## 4. Chemical Inhibition of Nuclear Exporter Function in Cancer

XPO1 is the exporter of several important genome surveillance proteins and tumor suppressors and some of these proteins play a prominent role in cancer suppression. Majority of TSPs and genome surveillance proteins reside in the nucleus of cell, bind to DNA in a sequence-specific manner and induce transcription of other surveillance proteins. Unusual export of these nuclear TSPs renders them ineffective. Therefore, targeted inhibition of nuclear exporter XPO1 becomes an attractive therapeutic strategy. Here we discuss several XPO1 inhibitors that have shown to exert potent anti-tumor activity in pre-clinical pancreatic cancer models as well as in cancer patients in clinic.

### 4.1. Natural Products with XPO1 Inhibitory Activity

Leptomycin B (LMB) was the first XPO1 inhibitor isolated from natural sources. LMB is a secondary metabolite produced by *Streptomyces* spp. and was originally studied for its anti-fungal properties and fission elongation in yeast saccharomyces pombe [42]. LMB covalently binds to NES recognizing Cys528 residue of XPO1, alkylates it and blocks its nuclear export function [43]. Due its XPO1 inhibitory effects, LMB was also shown to be a potent cell cycle arrest inducer and apoptosis inducer in cancer cells [44]. Mechanistically, LMB treatment has been shown to result in the nuclear retention of several TSPs in the cell nucleus and this has been attributed to its anti-cancer effects. Encouraged by these mechanistic studies, there are several publications that show the anti-tumor potential of LMB in a number of pre-clinical xenograft models including pancreatic cancer. These studies also led to a Phase I clinical trial in which the safety and efficacy of LMB (trade name Elactocin) was evaluated in patients with cancer [45]. Unfortunately, due to the covalent and irreversible binding nature of LMB it proved to be too toxic to patients on this trial. This clinical trial was halted midway and since then LMB has remained an in vitro tool compound for proof of concept studies only.

Two natural products Plumbagin and Curcumin have also been shown to have XPO1 inhibitory potential. Plumbagin is a napthoquinone that is derived from Plumbago zeylanica. It has been well studied for its anti-cancer effects in vitro and in vivo [46]. Liu et al. used mass spectroscopy to demonstrate that plumbagin could specifically bind to the wild type Cys528 amino acid (not the mutant form) leading to blockade of the nuclear export function [47]. However, plumbagin by virtue of its pleiotrophic effects has several off target activities and could not be developed further as an anti-cancer agent in the clinical setting.

Curcumin that is derived from Turmeric is one of the most well studied natural product derivative for its anti-inflammatory and anti-cancer properties [48]. Niu and colleagues utilized a nuclear export functional assay, to demonstrate a global shift in cytoplasmic proteins into the nucleus when treated with curcumin and its analog dibenzylideneacetone (DBA) [49]. More significantly, their mass spectroscopy studies showed that the binding mechanism of curcumin to XPO1 was very similar to that of LMB, CBS9106 and plumbagin, i.e., Cys528 amino acids in vivo. They also predicted through computational modeling that curcumin could dock into the hydrophobic pocket of XPO1 through complementarity and putative molecular interaction assumptions. As with other known XPO1 inhibitors, curcumin was shown to act as the Michael acceptor enabling a Michael addition type reaction with Cys residue of XPO1. Despite such strong mechanistic studies supporting its XPO1 inhibitory effects, curcumin has poor bioavailability and several off-target activities. Therefore, it is very hard to predict whether agents like curcumin or plumbagin could be further developed into clinically viable therapies especially in the context of XPO1 inhibition.

Several additional natural product derivatives have been shown to block XPO1 nuclear export function. Wach and colleagues showed that (R)-goniothalamin could block XPO1 mediated nuclear export at sub-micro molar concentrations [50]. The (R)-goniothalamin was used as the starting point for rational drug design of novel small molecule XPO1 inhibitors via an enantioselective Cr(III) catalyzed hetero Diels-Alder reaction and a Sonogashira coupling. This approach led to the development of several analogs with potent XPO1 inhibitory activity. Although such compounds have not been investigated in early phase clinical studies, they certainly show the potential to be developed as an alternative to LMB. Similarly, the search of G(2) checkpoint inhibitors by phenotypic cell-based assay (by screening a library of plant extracts) led to the identification of pyrones Z-Cryptofolione and Cryptomoscatone D2 with nuclear export inhibitory properties [51]. These pyrones could block nuclear export function and induce G(2) arrest in cancer cells. On similar lines, Cautain and colleagues performed high throughput screening to identify XPO1 inhibitors in microbial origin chemical libraries. Their library included extracts from fungi, actinomycetes, and unicellular bacteria. Using a cell-based imaging assay (U2nesRELOC), these authors identified fungal metabolite MDN-0105 with low micro anti-cancer activity and TSP nuclear retention abilities [52].

### 4.2. Chemically Synthesized XPO1 Inhibitors

During the initial years of small molecule inhibitor discovery, the NES of HIV-1 Rev was also identified to be targeted by XPO1 [53]. HIV-1 Rev is a protein that is an essential regulator of the HIV-1 mRNA expression and has been shown to promote the export of unspliced and partially spliced mRNA. Working in this direction Daelemans and colleagues identified a low molecular weight compound PKF050-638 that acted as an inhibitor of the XPO1-mediated Rev nuclear export [53]. By using a quantitative in vitro CRM1-NES cargo-binding assay, they showed that PKF050-638 could block XPO1-NES interaction. Nevertheless, PKF050-638 remained an in vitro tool compound for HIV-1 Rev transport mechanism studies.

Screening of libraries for chemicals with cell cycle arrest inducing properties led to the discovery of CBS9106 [54]. This compound was also shown to be a potent inhibitor of XPO1. Similar to LMB, CBS9106 blocks XPO1 export function by binding to the cys528 amino acid in the NES recognizing domain, retains TSPs in the nucleus and induces cell cycle arrest and apoptosis in cancer cells. However, biotinylation experiments demonstrated that unlike LMB, CBS9106 does not bind irreversibly to XPO1. Such reversible binding to XPO1 was projected to be a safer and less toxic approach to block XPO1 compared to the predecessor compound LMB. Despite these promising pre-clinical results the clinical utility of CBS9106 has not been investigated till date. Another reversible XPO1 inhibitor S109 (also a cys528 binder) has been shown to be effective in suppressing the growth of several solid tumors [55,56]. S109 could suppress XPO1 protein levels through proteasomal turnover.

Recently, Ali Cagir and colleagues have generated a series of compounds that are 6-(naphthalen-1-yl) substituted 5,6-dihydro-2H-pyran-2-one derivatives showing promising antiproliferative activities in variety of cancer cell lines and spheroid 3D culture models [57]. Termed Klavuzones, these agents were shown to have strong XPO1 inhibitory activity. Although very potent in in vitro assays, the activity of these novel XPO1 inhibitors in xenograft models is not known.

### 4.3. Specific Inhibitor of Nuclear Export (SINE)

The SINE compounds are a unique class of XPO1 inhibitors developed at Karyopharm Therapeutics Inc. that have shown notable activity in pre-clinical and clinical studies [58]. SINE compounds [KPT-185, KPT-257, KPT-271, KPT-330 (selinexor), KPT-8602 (Eltanexor)] like LMB, block the nuclear export function by binding to the Cys 528 amino acid in XPO1 [59]. However, unlike LMB that is a covalent and irreversible inhibitor, SINE compounds bind to Cys528 in a slowly reversible fashion. SINE compounds have been extensively examined in a broad spectrum of tumor models (ranging from solid tumors to hematological malignancies). These compounds show low nanomolar IC_50s_ against cancer cells and have normal cell sparing properties. The anti-tumor activity of these compounds either alone or in combination with respective standard of care therapeutics have been documented in prostate cancer, lung cancer, breast cancer, glioblastoma, colon cancer, renal cell carcinoma, hepatocellular carcinoma, kidney cancer, thymic cancer, non-Hodgkin’s lymphoma, CLL, AML, ALL and others [60,61,62,63,64,65,66,67,68,69,70,71,72,73,74,75]. The lead compound selinexor is fairly well tolerated and has been shown to suppress tumor growth in xenograft models (when given at ~15 mg/kg every other day for three weeks schedule). Cys528 binding for most of the previous XPO1 inhibitors were confirmed by site-directed mutagenesis. However, for SINE compounds more specific assays such Crispr/Cas9 genome editing have been used to demonstrate the Cys528 specific binding [76].

Unlike other XPO1 blockers, SINE selinexor and related analog altenexor (KPT-8602, the next generation SINE compound) are the only agents that have completed successful Phase I and Phase II studies. A search on the ClinicalTrials.gov website using keyword “selinexor” reveals 60 clinical studies in several tumor types (https://clinicaltrials.gov/ct2/results?cond=&term=selinexor&cntry=&state=&city=&dist=). More than 1000 patients have been exposed to selinexor in these trials combined. The published results of several of these trials have shown evaluable response in patients and support the safety and efficacy of selinexor for the treatment of cancer [77,78,79,80,81,82,83,84,85,86].

## 5. Activity of SINE in Pancreatic Cancer

In view of the role of aberrant XPO1 expression in pancreatic cancer subsistence, the anti-tumor activity of SINE were studied in several pre-clinical and Phase Ib clinical trial. Our group was among the first to investigate the activity of SINE analogs in multiple pancreatic cancer cell lines, sub-cutaneous xenograft and orthotopic animal models [87]. SINE analogs (KPT-185, KPT-251, KPT-276 and KPT-330/selinexor) demonstrated broad specificity and low nanomolar IC_50_ in a spectrum of pancreatic cancer cell lines. SINE could effectively block pancreatic cancer cell proliferation, induce apoptosis and retain important tumor suppressor proteins in the nucleus. The drug was effective across a panel of pancreatic cancer cell lines (such as BxPc-3, AsPC-1, MiaPaCa-2, L3.6pl, Colo-357, Panc-1, Panc-28) irrespective of their K-ras or p53 mutational status. Site-directed mutagenesis studies highlighted the role of nuclear retention of several functional TSPs such as FOXO3a, p73, Hsp90, PAR-4 among others as part of SINE mechanism of action. Most significantly, we observed strong oral anti-tumor activity in pancreatic cancer cells derived sub-cutaneous and orthotopic tumor models. Interestingly, molecular analyses of the residual tumors showed nuclear retention of several important TSPs alongside the activation of apoptotic signaling in situ, thereby confirming the in vitro mechanism of action. This initial report was further expanded to evaluate the anti-metastatic, anti-invasion activity of SINE in pancreatic cancer cellular and animal tumor models. Our studies showed that SINE compounds could block cell invasion and migration through the nuclear retention of the FBOX protein FBW7 resulting in the inhibition of notch signaling [88]. These in vitro studies could be recapitulated in vivo in residual tumors where we showed enhancement in FBW7 and notch inhibition post oral SINE treatment in vivo.

Supporting our initial findings, results from independent groups verified that SINE could enhance the cytotoxic effects of gemcitabine in pancreatic cancer cell lines and xenografts [89]. The combination of SINE compounds with gemcitabine was found to be synergistic [combination index (CI) < 1)]. Encouraged by this work, we further explored combination of clinical grade SINE selinexor with commonly used regimen gemcitabine-nab-paclitaxel in pancreatic cancer cellular, stem cell and patient-derived (Pdx) tumor models [41]. Our results reveal that SINE-gemcitabine-nab-paclitaxel could drastically reduce the growth of pancreatic cancer cell lines, suppress spheroid formation in CSCs, and block CSC xenograft growth. Most importantly SINE-gemcitabine-nab-paclitaxel could drastically suppress the growth of highly resistant patient-derived tumors (SINE given at 15 mg/kg every other day for three weeks).

As mentioned above, pancreatic tumors are heterogeneous and tend to harbor several types of cells with differential sensitivities. Studies have linked such therapy resistance to the presence of stem-like cell population with high expression of CD24;CD133;EpCAM. These stem-like cells or CSCs can grow in long term culture conditions as spheroids, can form tumors when inoculated at low numbers and can undergo epithelial-to-mesenchymal transition (EMT). Therefore, strategies that can hit bulk tumor as well as these resistant cells and simultaneously prevent EMT are needed against pancreatic cancer. Interestingly in a snail over-expressing human mammary epithelial cell line with EMT phenotype (HMEC-snail) XPO1 RNAi could prevent stemness, reverse EMT and also prevent spheroid formation [90]. Our recent work in pancreatic-derived CSCs is aligned to these previous findings. MiaPaCa-2 derived CSCs demonstrate higher expression of XPO1 compared to parent cell line. Further, XPO1 RNAi could suppress spheroid formation in vitro and also suppress the growth of CSC-derived tumor xenograft [41]. These studies indicated that XPO1 inhibition can become a viable strategy against stem-like and EMT harboring cells.

Our group also evaluated the global changes in microRNA expression post selinexor treatment. We have demonstrated that inhibition of XPO1 by the selinexor increases miR-145 expression in pancreatic cancer cells resulting in the decreased cell proliferation migratory capacities. These results could be re-capitulated using forced expression of miR-145 in pancreatic cancer cell lines. Further mechanistic experiments revealed that selinexor or analog treatment resulted in the down-regulation of known miR-145 targets genes including EGFR, MMP1, MT-MMP, c-Myc, Pak4 and Sox-2. In this analysis, we also observed selinexor mediated re-activation of two additional tumor suppressive miRNAs miR-34c and let-7d that promoted p21WAF1. Collectively these results proved that targeted inhibition of the nuclear export machinery could restore tumor suppressive miRNAs in pancreatic cancer.

Building on these existing findings there are several combination studies currently in the pipeline. Selinexor is being studied in combination with gemcitabine; gemcitabine-nab-paclitaxel, extracellular receptor kinase (ERK) inhibitors, PARP inhibitors, EGFR targeted therapies, immune-checkpoint inhibitors, MEK and Akt inhibitors.

As mentioned above, XPO1 inhibition can inhibit several critical pathways that promote pancreatic cancer progression and therapy resistance. This was shown in several pre-clinical cellular models, 3D models (CSCs) and patient derived xenograft [41]. Encouraged by so many different pre-clinical evidences this work led to the initiation of a Phase Ib/II clinical study entitled Selinexor, Gemcitabine Hydrochloride, and Paclitaxel Albumin-Stabilized Nanoparticle Formulation in Treating Patients with Metastatic Pancreatic Cancer (Clinicaltrial.gov identifier NCT02178436). In this partially randomized phase Ib/II trial the primary goals are to evaluate the side effects and best dose of selinexor when given together with gemcitabine hydrochloride and paclitaxel albumin-stabilized nanoparticle formulation and to see how well they work in treating patients with metastatic pancreatic cancer. At present the Phase I portion of this trial is complete and interim analysis to determine whether the Maximum Tolerated Dose (MTD) has been reached is ongoing.

It is recognized that pancreatic tumors harbor a dense stroma which hinders proper drug penentrance. Such low drug delivery has been linked to the lack of efficacy of commonly used chemotherapeutics such as gemcitabine or gemcitabine-nab-paclitaxel. One can argue that the pancreatic tumor-associated stroma may hinder appropriate delivery of selinexor to the tumor site. In this direction, using in vivo mouse studies, XPO1 occupancy could be measured in tumors (non-pancreatic model) and was found to be dose-dependent, with >90% target saturation at 10 mg/kg (which is equivalent to ~50 mg flat dose in humans) [91]. Drug-target occupancy was measured in a dose-response time course and full occupancy occurred by 6 h at all doses. The duration of occupancy was dose-dependent, where 10–15 mg/kg in mice (equivalent to ~50–75 mg human flat dose) was necessary to maintain XPO1 occupancy up to 48 h post-dose. These findings confirm the selinexor RP2D of 60 mg (a dose that is being given in the Phase Ib/II pancreas trial) is sufficient for achieving target occupancy and inhibition up to 48 h. Nevertheless, the occupancy of selinexor needs to be further analyzed in pancreatic tumors and such work is ongoing.

## 6. Conclusions

Pancreatic cancer remains a deadly disease with single digit five-year survival statistics. There is a dire need for novel therapeutics to tame this highly recalcitrant malignancy. Pancreatic cancer is recognized to frequently harbor K-ras mutations. However, till date, this has remained a non-druggable target. There are a limited number of tumor suppressors, such as p53, SMAD, and p16 that are found to be mutated in pancreatic cancer as well. However, aside from these few, the majority of other tumor suppressors remain wild type. Nevertheless, these tumor suppressors are found to be functionally inactivated through several post-translational mechanisms. One plausible mechanism for tumor suppressor protein inactivation is their excessive nuclear export by the nuclear exporter XPO1. XPO1, often over-expressed in pancreatic cancer, mislocalizes the wild type tumor suppressors to the wrong cellular compartment, leading to their functional inactivation. Therefore, blocking of the nuclear exporter through specific inhibitors of nuclear export becomes a viable therapeutic strategy. As discussed in this review, the initial proof of concept studies clearly show that targeting the nuclear export machinery could result in the restoration of tumor suppressor functions, prevent stemness, and reverse EMT in pancreatic cancer. Specific inhibitors of nuclear export have entered clinical trials for pancreatic cancer. These developments bring hope that interfering with the nuclear protein transport machinery may one day become a viable therapeutic strategy for this devastating and deadly malignancy.
